# Supplementary material for: Complex‐centric proteome profiling by SEC‐SWATH‐MS
Source: Mol Syst Biol. 2019 Jan 14;15(1):e8438. doi: 10.15252/msb.20188438 (PMC6346213; doi:10.15252/msb.20188438)
Supplement: Supplementary file 8 — Dataset EV7 [file MSB-15-e8438-s008.zip › feature_plots_string/P16144.pdf]

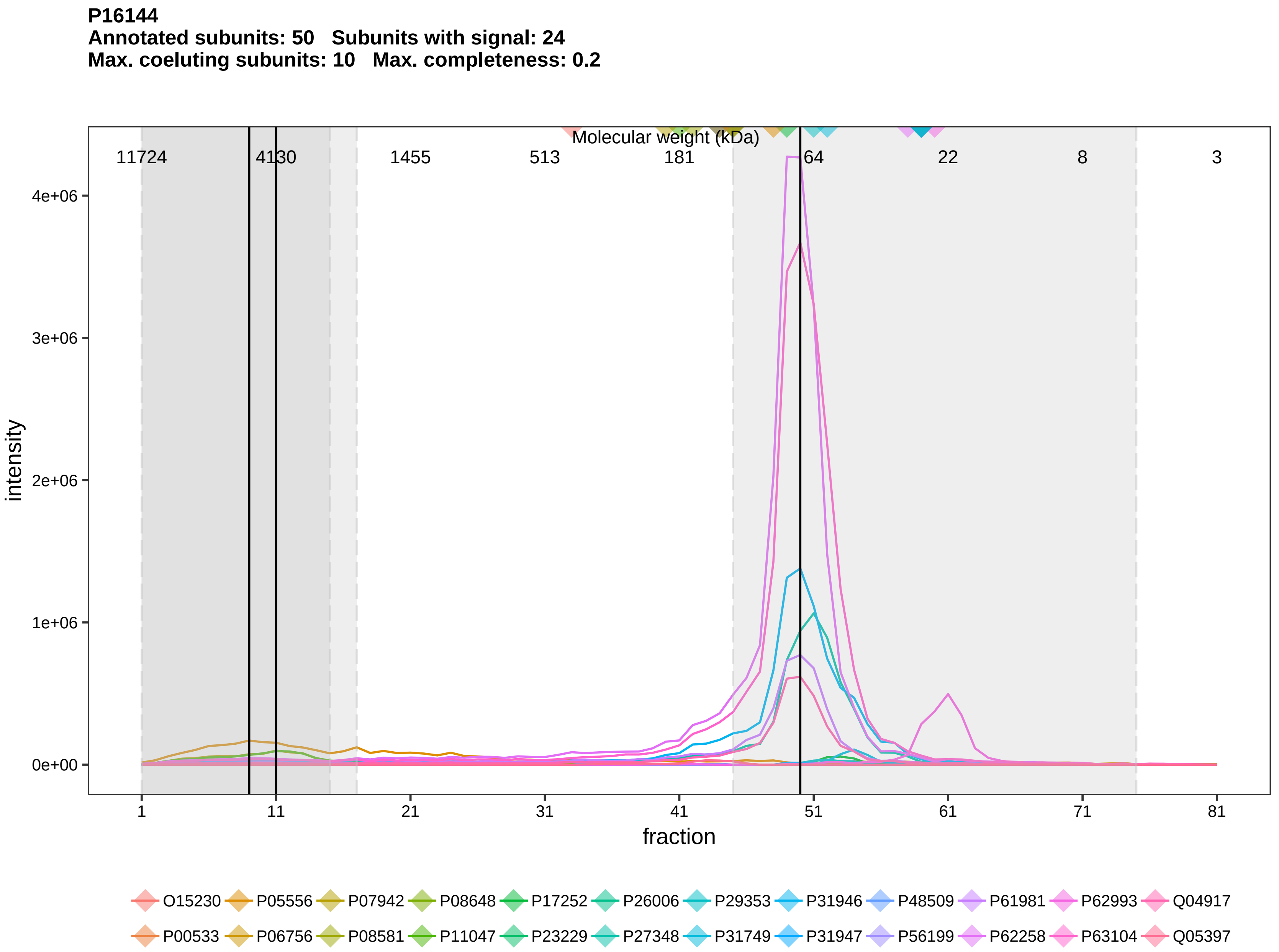
P16144
Annotated subunits: 50 Subunits with signal: 24
Max. coeluting subunits: 10 Max. completeness: 0.2
Molecular weight (kDa)
11724
4130
1455
513
181
64
22
8
3
intensity
4e+06
3e+06
2e+06
1e+06
0e+00
fraction
1
11
21
31
41
51
61
71
81
O15230
P05556
P07942
P08648
P17252
P26006
P29353
P31946
P48509
P61981
P62993
Q04917
P00533
P06756
P08581
P11047
P23229
P27348
P31749
P31947
P56199
P62258
P63104
Q05397
